# Physiological modeling, tight glycemic control, and the ICU clinician: what are models and how can they affect practice?

**DOI:** 10.1186/2110-5820-1-11

**Published:** 2011-05-05

**Authors:** J Geoffrey Chase, Aaron J Le Compte, J-C Preiser, Geoffrey M Shaw, Sophie Penning, Thomas Desaive

**Affiliations:** 1 Department of Mechanical Engineering, Centre for Bio-Engineering, University of Canterbury, Christchurch, Private Bag 4800, New Zealand; 2 Department of Intensive Care, Erasme University Hospital, B1070 Brussels, Belgium; 3 Department of Intensive Care, Christchurch Hospital, Christchurch, 8054, New Zealand; 4 Cardiovascular Research Centre, Universite de Liege, B4000 Liege, Liege, Belgium

## Abstract

Critically ill patients are highly variable in their response to care and treatment. This variability and the search for improved outcomes have led to a significant increase in the use of protocolized care to reduce variability in care. However, protocolized care does not address the variability of outcome due to inter- and intra-patient variability, both in physiological state, and the response to disease and treatment. This lack of patient-specificity defines the opportunity for patient-specific approaches to diagnosis, care, and patient management, which are complementary to, and fit within, protocolized approaches.

Computational models of human physiology offer the potential, with clinical data, to create patient-specific models that capture a patient's physiological status. Such models can provide new insights into patient condition by turning a series of sometimes confusing clinical data into a clear physiological picture. More directly, they can track patient-specific conditions and thus provide new means of diagnosis and opportunities for optimising therapy.

This article presents the concept of model-based therapeutics, the use of computational models in clinical medicine and critical care in specific, as well as its potential clinical advantages, in a format designed for the clinical perspective. The review is presented in terms of a series of questions and answers. These aspects directly address questions concerning what makes a model, how it is made patient-specific, what it can be used for, its limitations and, importantly, what constitutes sufficient validation.

To provide a concrete foundation, the concepts are presented broadly, but the details are given in terms of a specific case example. Specifically, tight glycemic control (TGC) is an area where inter- and intra-patient variability can dominate the quality of care control and care received from any given protocol. The overall review clearly shows the concept and significant clinical potential of using computational models in critical care medicine.

## The critically ill patient

Critically ill patients can be defined by the high variability in response to care and treatment. In particular, variability in outcome arises from variability in care and variability in the patient-specific response to care. The greater the variability, the more difficult the patient's management and the more likely a lesser outcome becomes. Hence, the recent increase in importance of protocolized care to minimize the iatrogenic component due to variability in care. Recent articles [1,2] have noted that protocols are potentially most applicable to groups with well-known clinical pathways and limited comorbidities, where a "one size fits all" approach can be effective. Those outside this group may receive lesser care and outcomes compared with the greater number receiving benefit.

**Figure 1:**
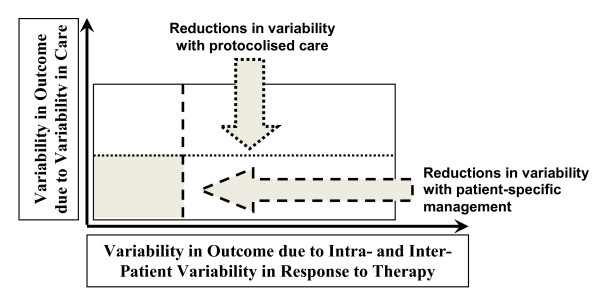
**Variability in outcome of the critically ill patient defined by variability in response to therapy and variability in care**. Shaded area defines the target zone for patient-specific care.

Figure 1 defines this problem in terms of variability in care that protocolized care can reduce, and a different, potentially less reducible, component due to inter- and intra-patient variability in response to treatment. The larger the area, the more difficult the patient can be to manage. Thus, protocolized care reduces only the nonpatient portion of this diagram. Equally, those whose clinical pathway is "straightforward" and can benefit most from protocolized care are likely to have limited inter- and intra-patient variability in response to treatment. Hence, the smallest, least variable case is one in which intra-patient response is reduced or managed in a patient-specific fashion, thus separating the final area into several smaller ones. A focus of this paper is that the model-based methods discussed here can provide patient-specific care that is robust to these intra- and inter-patient variabilities.

This issue is evident in many areas of care. For example, why are the complications of diabetes and therapeutic anticoagulation a leading cause of death or iatrogenic harm when they are amongst the most highly researched and understood fields in medicine? A PubMed search using the key words "diabetes mellitus" and "anticoagulation" returned 19,008 and 288,774 references, respectively, and a Google search multiplied these numbers to 1.14 M and 9.48 M pages. The collective experience of the drugs used in these conditions also is enormous; insulin, heparin, and warfarin were first used in humans more than 89, 76, and 57 years ago, respectively, and yet despite huge knowledge and experience, management of these conditions is fraught with problems.

What has led to this paradox? If, for example, managing diabetes was as straightforward as popping a few tablets or a daily insulin injection, doctors and patients would not still be struggling to get this right. Unfortunately, patients with diabetes have a widely variable clinical response, both within and between individuals, which often leaves clinicians unsuccessfully grappling with these nonlinear behaviors and responses. The randomized controlled trial (RCT) is regarded as the most reliable instrument on which to base treatment selection. However, recommendations from RCTs are based on overall cohort responses, not individual responses, and therefore cannot provide the necessary patient-specific therapeutic guidance, particularly when variability can have a major impact in titrating treatment.

We examine and review a new, emerging therapeutic approach that provides for individualized care that accounts for intra- and inter-patient variability within an overall protocolized and evidence-based framework. This review is done with reference to the management of glucose intolerance and diabetes in critically ill patients, but the overall approach is readily generalizable to other areas of intensive care medicine.

## Physiological and clinical problem

Critically ill patients often experience stress-induced hyperglycemia and high insulin resistance [3-5] associated with increased morbidity and mortality [6-8]. Strong counter-regulatory (stress) hormone and proinflammatory immune responses lead to extreme insulin resistance and hyperglycemia, often exacerbated by high carbohydrate nutritional regimes and (relative) insulin deficiency. Inter- and intra-patient variability over different patients and as patient condition evolves make providing consistently tight glycemic control (TGC) across every individual patient a significant challenge, despite the growing use of protocolized care approaches.

This article uses TGC to present how computer models can be used at the bedside, within protocolized care, to provide patient-specific care and thus reduce the impact of intra- and inter-patient variability and provide care (within the shaded lower corner of Figure 1). TGC is a particularly apt example for model-based methods, as intra- and inter-patient variability in response to insulin can be extreme, leading to significant difficulty in providing safe and effective control [9].

In particular, recent randomized trials of TGC have failed to repeat promising early results [10-12]. Equally, reduced outcomes due to hyperglycemia, hypoglycemia (if control is poor), and glycemic variability [13,14], and the overall physiological basis in inflammatory and oxidative stress responses are increasingly understood [15-17]. Thus, it seems increasingly clear that protocolization of care alone has not been able to reduce the variability in patient outcomes and that patient-specific solutions that manage inter- and intra-patient variation may be required to determine if TGC offers significant benefit. Hence, this review examines (physiological) model-based methods for TGC as a case example of the patient-specific solutions that are possible and the potential of these methods to improve care.

## A series of questions

This review takes the reader through mathematical models in the context of TGC based on a series of clinically focused questions.

### What is a mathematical model? Physiological relevance and representation

A mathematical model is a mathematical description of reality. In physiology, such a model underlies a certain number of assumptions about the physical, chemical, and biological processes involved. These mathematical models may vary significantly in their complexity and their objectives. They can range from relatively simple lumped-compartment models [18-20] to very complex network representations and finite element models of several million degrees of freedom [21,22].

**Figure 2:**
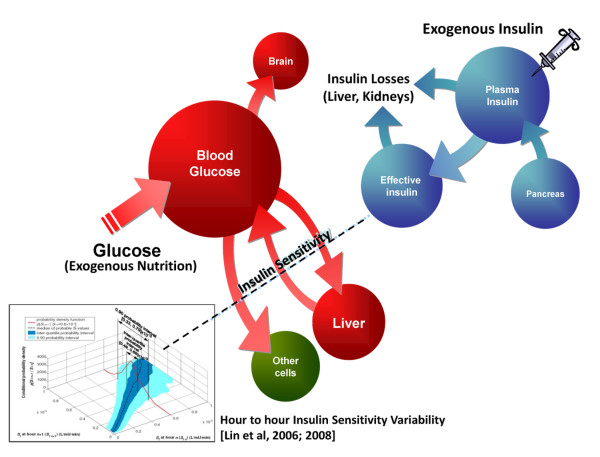
**Relevant physiology required to create effective models of human metabolism for the critically ill patient**. Insulin sensitivity is a whole body parameter representing is the average of the insulin resistance of each particular organ, which are all differentially regulated in stress conditions, and thus the dashed line indicates insulin-mediated uptake. Its value is patient-specific and can vary hourly [48,73].

For model-based TGC, the models should capture the fundamental underlying physiology as illustrated schematically in Figure 2. In particular, they should capture the transport of exogenous insulin, the production of endogenous insulin, the appearance of endogenous and exogenous carbohydrate as blood glucose, and, critically, both insulin-mediated and insulin-independent uptake of glucose. In addition, insulin-mediated uptake must have the ability to capture inter- and intra-patient variability in the time-varying insulin resistance observed in these patients. The model structure and physiological relevance of Figure 2 is detailed in several references [23,24] and in the appendix in Additional File 1 {AU Query: please cite the appendix as Additional file 1} with TGC specific modeling details for the interested reader.

In the critical care arena, the use of in silico physiological models is only emerging. However, there are already model-based or model-derived applications for managing sedation [25,26], cardiovascular diagnosis and therapy [27,28], mechanical ventilation [29,30], and the diagnosis of sepsis [31,32]. Particular to TGC, there are already some attempts at modeling for both understanding and implementing TGC [23,33-42], with a review of many in [43].

### What can a model do? Capabilities and limitations

All models have different uses or goals. A model may be used to describe, interpret, predict, or explain [18,19] a physiological process. Real capabilities depend on the chosen degree of approximation, based on a combination of the knowledge of the physiological processes involved and implementation goal.

However, a model definition is not enough. Model parameter values must be assumed from clinical data or reports, or identified (mathematically) from clinical data. These values determine whether the model is generic to a population or (more) patient-specific with parameters identified from a particular patient's data. In reality, most models are a mixture of both approaches, where patient-specific parameters are identified for those parameters critical to the application.

However, once identified, patient-specific models in particular offer a range of potential opportunities, including, for TGC, the:

• Simulation of so-called virtual patients [41,44-48] to design [33,41], analyze [49,50], or optimize glycemic control methods.

• Implementation at the bedside for patient-specific care in which patient-specific model parameters are identified in real-time to guide care [34-36,40,47,51-53].

Equally, metabolic models can be used with patient data to investigate a range of physiological behaviors [54-56].

In intensive care, patient-specific metabolic model parameters also have been used as sepsis biomarkers because they can accurately reflect the inflammatory status of the patient and severity of illness [31,32]. These studies showed that model-based insulin sensitivity alone could provide 70-80% sensitivity and specificity in assessing sepsis compared with a control cohort, yielding a negative predictive value (NPV) greater than 99%, thus clearly identifying periods where antibiotic therapy was not necessary. Such an outcome thus uses model-based physiological insight not otherwise available to provide a novel, non-invasive diagnostic.

Similarly, model-based insulin sensitivity has been used to assess the impact of glucocorticoid therapy on glycemic control [57]. In particular, it has been thought that glucocorticoid therapy would significantly increase insulin requirements in TGC based on the results of studies showing significantly increased insulin resistance when given to healthy individuals. However, this modeling showed the effect to be 5-10 times smaller in ICU patients, to be highly patient-specific depending on patient status, and to (overall) have very little impact on TGC dosing requirements, as a result. The ability to discern patient-specific impacts at the bedside using the model can provide significant insight.

Finally, TGC models can be used to assess the quality of control achieved clinically relative to other protocols using virtual patients [24,33,46,50]. In Suhaimi et al [50] the multi-center Glucontrol trial [12] protocol was evaluated versus the control achieved with the Specialized Relative Insulin and Nutrition Titration (SPRINT) [58] protocol. The model and analysis yielded clear directions on protocol compliance and the importance of understanding nutrition delivery in the provision of TGC. It also was able to show a surprising similarity in the inter- and intra-patient metabolic variability of critically ill patients between the centers and studies compared.

Finally, physiologically relevant computer models have a longer, similar history in the broader diabetes field, primarily for research to gain patho-physiological insight rather than direct use in controlling glycemia [18,54,55,59-64].

All models have limitations. Limited bedside data and the quality of the mathematical process used to find model parameters from data (identification method) can have a significant impact on identified parameter accuracy and model performance [24,64-66], as well as entailing specific assumptions [23,24,46,67]. In particular, models that are not physiologically relevant [37] or do not have all the necessary physiology relevant to the patient group to which it is applied [68-70] can yield inaccurate results. These studies failed to capture the enhanced glycemic production and reduced renal and hepatic clearances, the balance of which can dominate the overall metabolic behavior of the critically ill. Similarly, one can over-model a situation with too much complexity and create models that are not useful for implementation. As a result, their predictive ability and use in control was less effective. Such limitations must be rigorously quantified [23,57] to understand the quality of answer that any given model can provide.

### How do we know that a model is good? Prediction and validation

Making suitable assumptions and choosing a desired degree of approximation do not naturally generate a "good" model. Similarly, being able to find model parameters that ensure it fits a set of clinical data does not make a model valid, except to show that it can capture the dynamics observed clinically. It is critical to validate the model to determine if its performance is acceptable for its intended application.

For designing and/or implementing model-based TGC, where the model is directly used to provide patient-specific advice, it is necessary to ensure the models ability to:

• For design: predict the overall glycemic outcomes (median *and *variation) of patients and/or cohorts for a (simulated) protocol [44,46]

• For implementation: predict the glycemic outcome of a clinical intervention during a relevant 1- to 4-hour timeframe typical of TGC intervention frequencies [24,38,46,47,49,50,71,72].

These metrics define validity in its ability to capture patient-specific behaviors to a clinically acceptable level (approximately equivalent to measurement error). Errors thus reflect model limitations.

To date, only two ICU focused metabolic model structures have been validated with respect to individual patient-specific predictions (for implementation and design) [23,24,39,44,46]. Only one has been validated for cohorts [46].

The specific models in these studies define the critically ill patients by their time varying counter-regulatory and inflammatory status, as seen metabolically via their overall insulin sensitivity or metabolic balance that can vary hourly in acute cases, as illustrated in Figure 2. All other parameters were set at population constants following detailed parametric sensitivity studies based on assessing parameters impact on predictive performance [23,24,48]. Hence, the models provide median blood glucose prediction errors for specific interventions that are less than 3-4%. When an independent clinical protocol was simulated on virtual patients created the median cohort and patient glycemia and its variation were captured to within 3% and 5% respectively compared with the original clinical data (see [46] and appendix). Hence, validate the models and modeling approach, as well as show how they capture, through one main parameter, the metabolic dynamism of the critically ill patient.

### Why use models? Patient-specific insight and care from available data

The time-scale for decision making in the ICU ranges from 1-2 minutes in acute cases to hours for some therapies, such as mechanical ventilation or TGC. It often requires the synthesis of a wide range of patient-specific data across a number of monitors, assays, and physiological systems. Typically, clinicians apply their experience and intuition to make diagnoses and develop treatment plans, based on how they aggregate that data and how it fits their mental model of what they are observing. More specifically, they are using this data and a mental model to estimate occult physiological variables (i.e., make a diagnosis or determine patient state) and from that developing decisions for treatment. Given the range of experience, intuition, and mental models across clinicians, diagnosis is open to error and care can be quite variable.

A validated and relevant physiological model can create a more consistent, high-resolution physiological picture of the overall physiological system that also is potentially more accurate than the clinician's mental model. In particular, computer models and methods offer the ability to aggregate more data and to discern subtle trends in data that may otherwise be easily missed.

For model-based TGC, the patient-specific model variable that determines patient-specific state and response to therapy is the overall, whole body insulin sensitivity [42,48,73]. This value is itself the average of the insulin resistance of each particular organ, each of which is differentially regulated in stress conditions and sets the balance between insulin and nutrition inputs and outcome glycemia. However, given the variations in patient kinetics and levels of these inputs, it is very difficult, if not impossible, for a clinician to review these and arrive at an accurate assessment of its current value. But, without such a value, optimal dosing of insulin, including the effects of insulin saturation, for example, is not possible with any resolution.

Hence, the ability of a validated, physiologically relevant model to provide a patient-specific value and its potential variation in future offers unique insight and potential to optimize interventions that is not otherwise available [48,72,73]. Thus, validated, patient-specific models can test these insights and proposed treatments in silico, before application, improving safety. Because they use existing data and can predict accurately they offer the clinician a window on past and present behaviors, as well as a view of how to customize treatment for optimal future behaviors.

### What are the differences between computer-based, model-based, and model-derived TGC? The model, the implementation, and the level of patient-specificity

There are an increasing number of computer-based TGC protocols that are not model-based [74-79] and thus do not offer the same physiological insight or "picture." They are, more accurately, an extension of protocolized care in that they take a protocol and put it on the computer. Equally, such protocolized care provides a cohort-based approach that is consistent ("one size fits all") but not necessarily patient-specific. Thus, the main element that differentiates a model-based system is the use of a physiologically relevant, validated model to create a patient-specific picture of patient state and provide patient-specific ("one method fits all") advice.

A hybrid path uses what we denote "model-derived" protocols. The only current example of this approach is the SPRINT protocol [58]. This paper-based system was created and optimized in silico by using clinically validated models and virtual patients [33,80]. However, it provides patient-specific care, based on its design using the model, within the paper-based abstraction used to provide easy uptake in the ICU.

Hence, the critical difference is that model-based methods implicitly enforce a protocol, but, in their patient-specificity, translate the "one size fits all" approach of a fixed protocol to a "one method fits all" patient-specific form of care. For TGC these methods are already (increasingly) proven in both model-derived [44,58] and model-based [36,47,48,52,53] formats. Their success is due to their unique ability, when properly modeled and validated, to provide much better, real-time management of both intra- and inter-patient variability that typical non-model-based clinical protocols cannot and, as a result, provide a level of care that is beyond existing clinical protocols.

## Summary

Models and model-based methods have a lot to offer in a wide range of clinical areas in medicine, and in critical care specifically. Using TGC as an example, they can offer significant physiological insight into patient status and behavior that are not readily available at the bedside or part of the typical, clinical mental model. Hence, they enable the means to develop and implement "one method fits all" patient-specific approaches to diagnosis and care. Their ability to reduce the impact of intra- and inter-patient variability, within a protocolized framework that reduces variability in care, can improve care and outcomes for all patients. Hence, models and model-based methods represent an important area of potentially increasing significance to the practice of critical care medicine, and TGC in particular, in the coming years.

## Competing interests

The authors declare that they have no competing interests.

## Authors' contributions

JGC, GS, TD, JCP, SP, and ALC conceived and developed the review and written manuscript. All authors approved the final manuscript.

## Supplementary Material

Additional file 1 **Appendix: Metabolic System Model and Insulin Sensitivity (SI)**. This file contains a full description of the metabolic system model equations, their validation and physiological validity, the methods to identify the model-based insulin sensitivity (SI) parameter, its correlation to gold-standard tests, and, finally, the definition and application of stochastic models of model-based insulin sensitivity (SI). Click here for file
