# Supplementary material for: Physiological modeling, tight glycemic control, and the ICU clinician: what are models and how can they affect practice?
Source: Ann Intensive Care. 2011 May 5;1:11. doi: 10.1186/2110-5820-1-11 (PMC3224460; doi:10.1186/2110-5820-1-11)
Supplement: Additional file 1 — Appendix: Metabolic System Model and Insulin Sensitivity (SI). This file contains a full description of the metabolic system model equations, their validation and physiological validity, the methods to identify the model-based insulin sensitivity (SI) parameter, its correlation to gold-standard tests, and, finally, the definition and application of stochastic models of model-based insulin sensitivity (SI). [file 2110-5820-1-11-S1.DOC]

**Appendix: Metabolic System Model and Insulin Sensitivity (SI)**:

This appendix is designed to present the model and methods used in several referenced studies (e.g. [1-9]) in this paper. In particular, it addresses the model validation and validity, and, due to its rising concern in the field, it discusses the potential impact of sensor error on the results. The presentation is brief relying on a separate set of references (from the main article) given at the end of this appendix, which interested readers can use for explicit details on any aspect of this model and the methods used herein.

A-1: Model Definition:

A clinically validated computer model of the metabolic system [10] is used to identify [11] patient-specific, time-varying (hourly) insulin sensitivity (SI) every hour:

|  | (1) |
| --- | --- |
|  | (2) |
|  | (3) |

Where *G(t)* [mmol/L] is plasma glucose *I(t)* [mmol/L] is plasma insulin, *u_ex_(t)* [mU/min] is exogenous insulin input, basal endogenous insulin secretion is *I_B_* [mU/L/min], with *k_I_* representing suppression of basal secretion by exogenous insulin. Interstitial insulin is *Q(t)* [mU/L], with *k* [1/min] accounting for losses and transport. Body and brain weight are denoted by *m_body_* [kg] and *m_brain_* [kg]. Endogenous glucose clearance is *p_G_* [1/min] and time-varying insulin sensitivity is SI or (formally) SI(t) in Equation (1) [L/(mU.min)]. Finally, *V_I,frac_* [L/kg] is the insulin distribution volume per kg body weight and *V_I_* the resulting volume [L], and *n* [1/min] is the transport rate of insulin from plasma. Total plasma glucose input is *P(t)* [mmol/min], endogenous glucose production is *P_END_* [mmol/kg/min] and *V_G,frac_* [L/kg] represents the glucose distribution volume per kg body weight. *CNS* [mmol/kg/min] captures non-insulin mediated glucose uptake by the central nervous system. Michaelis-Menten functions model saturation, with *α_I_* [L/mU] for the saturation of plasma insulin disappearance, and *α_G_* [L/mU] for insulin-dependent glucose clearance saturation.

Figure A-1 shows this model (Figure 2 in the paper) schematically.

The insulin sensitivity SI can be identified hourly from blood glucose data along with the clinical insulin and nutritional inputs from all sources [12, 13]. Where the methods of these references are novel in the field (compared to e.g. [14-19]) and provide a unique, convex solution that other methods cannot. Hence, the values found for this critical parameter are guaranteed to be optimal and thus affected only by model resolution or sensor error.

SI is also the critical parameter in predicting the outcome of a nutrition and/or insulin intervention in this model, based on the definition above [2, 3, 12]. It represents the whole body balance of insulin and carbohydrate from all sources, and, in the highly inflammatory and counter-regulatory state of the critically ill patient it thus effectively captures patient status. Equally, it can vary with patient-status hour to hour, with larger acute changes or smaller gradual evolution. Thus, the identified parameters can be used to create models of this parameters evolution for cohorts or specific-patients that enable more optimal and robust dosing [20-23]. Figure A-1 shows this stochastic model in the overall model context and Figure A-2 shows its potential use in glycemic control

**Figure A-1**: Model schematic for Equations (1)-(3) showing the physiological compartments and clearances, as well as the appearance of exogenous insulin and carbohydrate, and their kinetic pathways. The stochastic model in the lower left shows how the insulin sensitivity (SI) can vary over time (hour to hour) thus affecting glycemic outcomes for a given insulin and/or nutrition intervention.

**Figure A-2**: Use of stochastic insulin sensitivity models to forecast likely BG outcomes for a given intervention, using the model of Equations (1)-(3). This approach allows the optimisation of care and its safety from hypoglycemia.

A.2: Model Validity and Validation:

The validity of the model and in particular the SI value identified is based on three types of studies and analyses:

- Correlation of the SI versus gold standard measures for whole body insulin sensitivity in the hyperinsulinemic euglycemic clamp (EIC), including its ability to measure changes in EIC derived insulin sensitivity after an intervention [24-26].
- Use of the model to predict the glycemic outcomes of an insulin and nutrition intervention [9, 23, 27] on retrospective data from the SPRINT [28] and Glucontrol [29] studies, as well as in similar predictive use in real-time TGC in the ICU [2-6] and NICU [20, 21] to guide therapy and optimise insulin dosing.
- A specific validation study [10] in which virtual patients [1, 12] are created from fitting this time-varying SI value to clinical data using novel integral-based methods [12], and then tested in their ability to predict the patient-specific and overall cohort glycemic outcomes when simulating another protocol using a matching cohort.

Results from these three types of validation are presented briefly below with relevant references to published literature for this model.

The model-based SI was fitted to data from 146 EIC tests [30] on 73 individuals before and after an intervention, including a control group. The SI marker correlation to the EIC derived ISI (insulin sensitivity index) was R = 0.99. Importantly, when analysing the change in ISI versus the change in SI before and after the intervention, the correlation was R = 0.94 [24, 26]. Hence, for the gold standard metric the model defined in Equations 1-3 is able to provide very high correlation to a gold standard metric as well as its change after an intervention, thus validating its ability to capture the fundamental insulin sensitivity dynamic. These results are shown in Figure A-3.


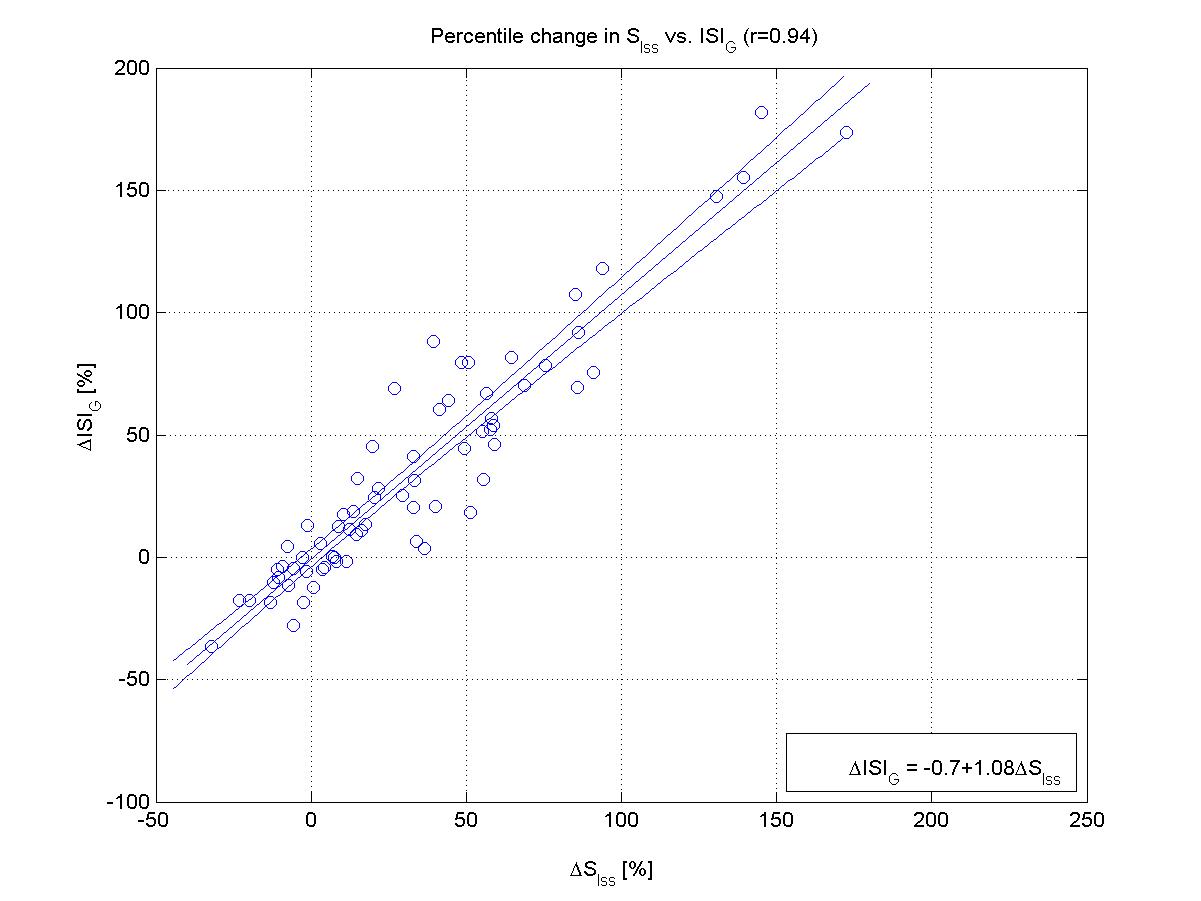

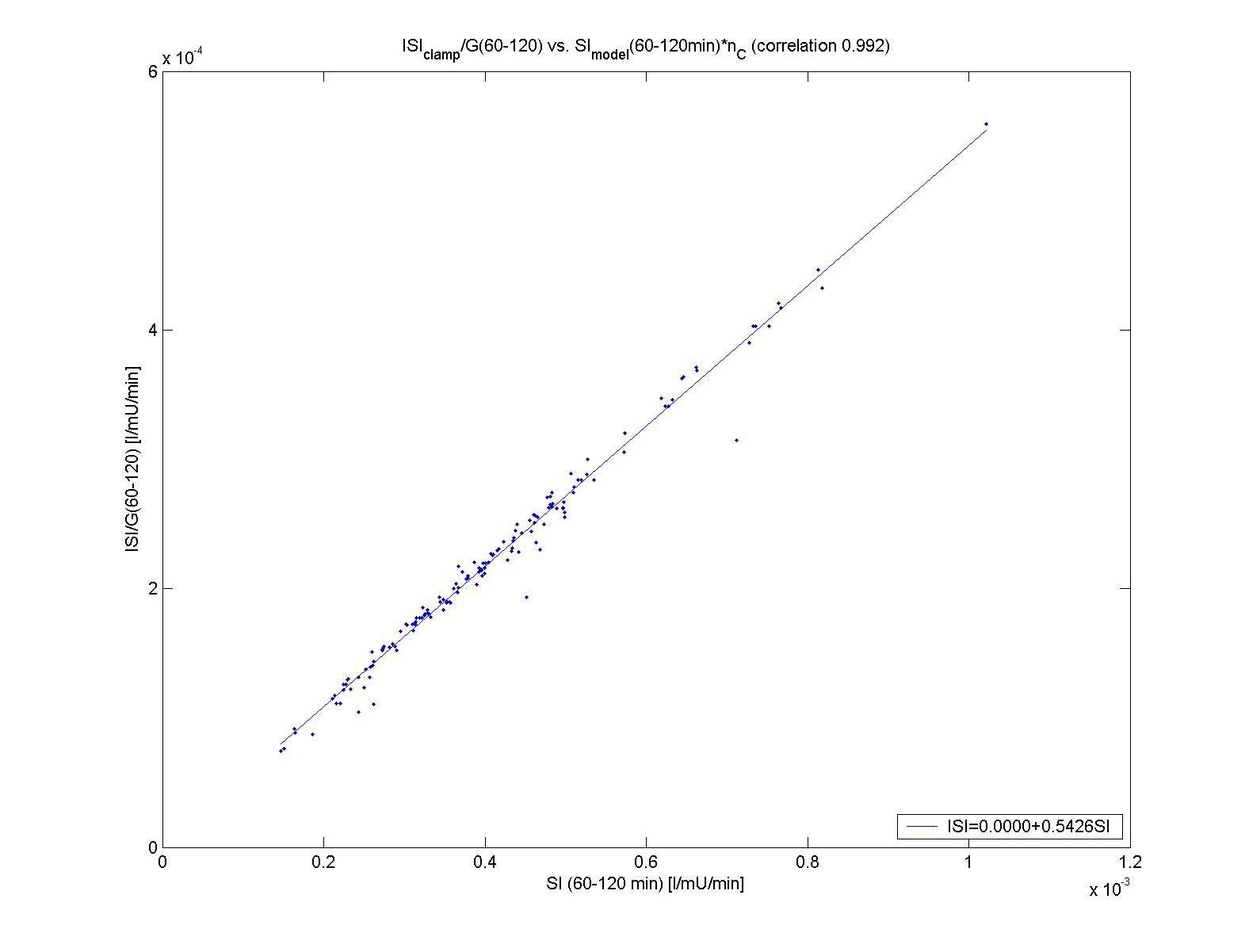


**Figure A-3**: Clamp study correlation showing how the model-based SI metric accurately captures gold standard assessment of insulin resistance, as well as its change over a series of interventions [24, 26].

For any model, the ability to predict the outcomes of an intervention are critical. Fitting only the SI metric to clinical data and then predicting forward using the clinically given intervention, the error between predicted outcome BG and the clinically recorded value is critical. Errors equal to or less than measurement error indicate optimum possible measurement performance. In several studies using data from ICU and NICU patients [9, 20, 27], this model, predicting 1-4 hours ahead, captures up to 40,000 such predictions with median errors between 3-8%, which is equivalent to the measurement errors of 7-10%. These studies have been done using data from SPRINT and from the Glucontrol trial, covering over 1100 patients and ~100,000 hours of data, as well as for NICU patients. Critically, the ability to predict, using only SI as a patient-specific parameter is thus entirely dependent on the validity of that parameter.

Equally, prediction errors can be a function of patient variability over the prediction time frame. Stochastic models of insulin sensitivity created from SPRINT or NICU data that measure this variability from the value one hour to the value the next have been used to test this model [21-23] using the method illustrated in Figure A-2. Predictions as above fall into expected IQR and 90% confidence intervals to within 1% of the expected number (e.g. 49% in a 50% wide IQR), even when considering cross validation and testing [23, 31]. These results indicate that the SI metric and model capture patient variations and the ability to predict the outcome of interventions to a level comparable to sensor error.

Finally, a full validation study was run using matching cohorts from the Glucontrol TGC trial and its Liege, Belgium centre [10]. Virtual patients were created form patient data for both the A and B arms of the trial, creating two sets of matching virtual patients. These patients were then simulated with both the A and B clinical protocols, creating both self and cross validations. Self validation tests model error in testing A virtual patients on the A protocol and then comparing to the group A clinical data, and similarly for the B group. Cross validation provides a guide as to the overall model quality in that it tests the B group on the A protocol and compares to the group A clinical data, thus testing whether the model and SI metric can capture glycemic outcomes for interventions independent of the data and treatment used to create the virtual patient. And, similarly for the A cohort on protocol B. Results for cohorts and median patients were within 1-10% across the cumulative distribution function of glycemic results. Similarly, the insulin interventions were also comparable. These results are shown in Figure A-4.

**Figure A-4**: Results from [10] on the validation of the model of Equations (1)-(3) using independent data from matched cohorts in the Glucontrol TGC trial. The results clearly show the ability to capture cohort (upper) and patient (lower) behaviour (median and variability). No other model is validated to this extent at this time.

The overall result of this study is a form of independent (crossover) validation that shows that this model and SI metric, and the methods used to find it, are able to accurately capture the dynamics of ICU patient. No other such complete validation exists in the literature for any similar model or virtual patient.

Thus, these sets of studies covering gold standard comparators, patient-specific predictive ability and an overall independent (cross) validation, serve to support the overall validity of the models and methods used in this article. Interested readers are directed to the references for further details.

A.3: Identification of SI and Impact of Sensor Error:

The hour to hour value of SI is identified from clinical data (BG, insulin given, nutrition and other dextrose or glucose given) for a specific patient. The method is a novel integral-based method that is convex and thus does not suffer well-known issues with local minima and non-optimal results found with other approaches (e.g. non-linear recursive least squares). The details of this method are in Hann et al [12, 13].

The glucose sensor used in SPRINT [28] were glucometers (Arkray Inc, Super Glucocard II) with assay errors of 7-12% (CV) depending on glucose level. Note that the lower value holds for the majority of measurements which were in the 4-8 mmol/L range (~90%), and because arterial blood was used, rather than capillary blood. Blood gas analysers would provide a lower error of 1-3%.

In identifying SI using integral-based methods, the BG data is integrated rather than differentiated, which is an important difference. Specifically, integration acts as a low pass filter and thus the impact of noise or random sensor error is significantly reduced. As a result, the SI values identified are globally optimal from the method and much less affected by sensor error than they would be using more traditional gradient based techniques [13].

Finally, for the analysis in this article, any offset in a given SI value (and resulting offset in the ΔSI) can be in either direction as the assay error is random and normally distributed. Hence, given the large numbers of hours in a large analysis, the central limit theorem supports the fact that any such errors will effectively cancel in the overall distributions presented. If data sets were smaller (< 100-300 data points), then sensor error and its impact on the identified SI values is potentially an issue for assessment via other statistical means such as Monte Carlo analysis.

**APPENDIX REFERENCES**:

1. Chase JG, Shaw GM, Lotz T, LeCompte A, Wong J, Lin J, Lonergan T, Willacy M, Hann CE: **Model-based insulin and nutrition administration for tight glycaemic control in critical care**. *Curr Drug Deliv* 2007, **4**(4):283-296.

2. Chase JG, Shaw GM, Lin J, Doran CV, Hann C, Robertson MB, Browne PM, Lotz T, Wake GC, Broughton B: **Adaptive bolus-based targeted glucose regulation of hyperglycaemia in critical care**. *Med Eng Phys* 2005, **27**(1):1-11.

3. Chase JG, Shaw GM, Lin J, Doran CV, Hann C, Lotz T, Wake GC, Broughton B: **Targeted glycemic reduction in critical care using closed-loop control**. *Diabetes Technol Ther* 2005, **7**(2):274-282.

4. Wong XW, Singh-Levett I, Hollingsworth LJ, Shaw GM, Hann CE, Lotz T, Lin J, Wong OS, Chase JG: **A novel, model-based insulin and nutrition delivery controller for glycemic regulation in critically ill patients**. *Diabetes Technol Ther* 2006, **8**(2):174-190.

5. Wong XW, Chase JG, Shaw GM, Hann CE, Lotz T, Lin J, Singh-Levett I, Hollingsworth LJ, Wong OS, Andreassen S: **Model predictive glycaemic regulation in critical illness using insulin and nutrition input: a pilot study**. *Med Eng Phys* 2006, **28**(7):665-681.

6. Chase JG, Wong X-W, Singh-Levett I, Hollingsworth LJ, Hann CE, Shaw GM, Lotz T, Lin J: **Simulation and initial proof-of-concept validation of a glycaemic regulation algorithm in critical care**. *Control Engineering Practice* 2008, **16**(3):271-285

7. Lonergan T, LeCompte A, Willacy M, Chase JG, Shaw GM, Wong XW, Lotz T, Lin J, Hann CE: **A Simple Insulin-Nutrition Protocol for Tight Glycemic Control in Critical Illness: Development and Protocol Comparison**. *Diabetes Technol Ther* 2006, **8**(2):191-206.

8. Lonergan T, Compte AL, Willacy M, Chase JG, Shaw GM, Hann CE, Lotz T, Lin J, Wong XW: **A pilot study of the SPRINT protocol for tight glycemic control in critically Ill patients**. *Diabetes Technol Ther* 2006, **8**(4):449-462.

9. Suhaimi F, Le Compte A, Preiser JC, Shaw GM, Massion P, Radermecker R, Pretty C, Lin J, Desaive T, Chase JG: **What Makes Tight Glycemic Control (TGC) Tight? The impact of variability and nutrition in 2 clinical studies**. *Journal of Diabetes Science and Technology* 2010, **4**(2):284-298.

10. Chase JG, Suhaimi F, Penning S, Preiser JC, Le Compte AJ, Lin J, Pretty CG, Shaw GM, Moorhead KT, Desaive T: **Validation of a model-based virtual trials method for tight glycemic control in intensive care**. *Biomed Eng Online* 2010, **9**:84.

11. Hann CE, Chase JG, Lin J, Lotz T, Doran CV, Shaw GM: **Integral-based parameter identification for long-term dynamic verification of a glucose-insulin system model**. *Comput Methods Programs Biomed* 2005, **77**(3):259-270.

12. Hann CE, Chase JG, Lin J, Lotz T, Doran CV, Shaw GM: **Integral-based parameter identification for long-term dynamic verification of a glucose-insulin system model**. *Comput Methods Programs Biomed* 2005, **77**(3):259-270.

13. Hann C, Chase J, Ypma M, Elfring J, Nor N, Lawrence P, Shaw G: **The Impact of Parameter Identification Methods on Drug Therapy Control in an Intensive Care Unit**. *The Open Medical Informatics Journal* 2008, **2**:92-104.

14. Cobelli C, Carson ER, Finkelstein L, Leaning MS: **Validation of simple and complex models in physiology and medicine**. *Am J Physiol* 1984, **246**(2 Pt 2):R259-266.

15. Cobelli C, Pacini G, Toffolo G, Sacca L: **Estimation of insulin sensitivity and glucose clearance from minimal model: new insights from labeled IVGTT**. *Am J Physiol* 1986, **250**(5 Pt 1):E591-598.

16. Carson ER, Cobelli C: **Modelling methodology for physiology and medicine**. *Academic Press Series in Biomedical Engineering* 2001:xiv, 421.

17. Cobelli C, Caumo A, Omenetto M: **Minimal model SG overestimation and SI underestimation: improved accuracy by a Bayesian two-compartment model**. *Am J Physiol* 1999, **277**(3 Pt 1):E481-488.

18. Hovorka R, Chassin LJ, Ellmerer M, Plank J, Wilinska ME: **A simulation model of glucose regulation in the critically ill**. *Physiol Meas* 2008, **29**(8):959-978.

19. Pillonetto G, Sparacino G, Cobelli C: **Numerical non-identifiability regions of the minimal model of glucose kinetics: superiority of Bayesian estimation**. *Math Biosci* 2003, **184**(1):53-67.

20. Le Compte A, Chase J, Lynn A, Hann C, Shaw G, Wong X, Lin J: **Blood Glucose Controller for Neonatal Intensive Care: Virtual trials development and 1st clinical trials**. *Journal of Diabetes Science and Technology (JoDST)* 2009, **3**(5):1066-1081.

21. Le Compte AJ, Lee DS, Chase JG, Lin J, Lynn A, Shaw GM: **Blood glucose prediction using stochastic modeling in neonatal intensive care**. *IEEE Trans Biomed Eng* 2010, **57**(3):509-518.

22. Lin J, Lee, DS, Chase, JG, Hann, CE, Lotz, T and Wong, XW: **Stochastic Modelling of Insulin Sensitivity Variability in Critical Care**. *Biomedical Signal Processing & Control* 2006, **1**:229-242.

23. Lin J, Lee D, Chase JG, Shaw GM, Le Compte A, Lotz T, Wong J, Lonergan T, Hann CE: **Stochastic modelling of insulin sensitivity and adaptive glycemic control for critical care**. *Comput Methods Programs Biomed* 2008, **89**(2):141-152.

24. Lotz TF, Chase JG, McAuley KA, Lee DS, Lin J, Hann CE, Mann JI: **Transient and steady-state euglycemic clamp validation of a model for glycemic control and insulin sensitivity testing**. *Diabetes Technol Ther* 2006, **8**(3):338-346.

25. Lotz T: **High Resolution Clinical Model-Based Assessment of Insulin Sensitivity**. Christchurch: University of Canterbury; 2007.

26. Lotz TF, Chase JG, McAuley KA, Shaw GM, Wong XW, Lin J, Lecompte A, Hann CE, Mann JI: **Monte Carlo analysis of a new model-based method for insulin sensitivity testing**. *Comput Methods Programs Biomed* 2008, **89**(3):215-225.

27. Lin J, Razak NN, Pretty CG, Le Compte A, Docherty P, Parente JD, Shaw GM, Hann CE, Geoffrey Chase J: **A physiological Intensive Control Insulin-Nutrition-Glucose (ICING) model validated in critically ill patients**. *Comput Methods Programs Biomed* 2011, **Epub ahead of print**.

28. Chase JG, Shaw G, Le Compte A, Lonergan T, Willacy M, Wong XW, Lin J, Lotz T, Lee D, Hann C: **Implementation and evaluation of the SPRINT protocol for tight glycaemic control in critically ill patients: a clinical practice change**. *Crit Care* 2008, **12**(2):R49.

29. Preiser JC, Devos P, Ruiz-Santana S, Melot C, Annane D, Groeneveld J, Iapichino G, Leverve X, Nitenberg G, Singer P *et al*: **A prospective randomised multi-centre controlled trial on tight glucose control by intensive insulin therapy in adult intensive care units: the Glucontrol study**. *Intensive Care Med* 2009, **35**(10):1738 - 1748.

30. McAuley KA, Williams SM, Mann JI, Goulding A, Chisholm A, Wilson N, Story G, McLay RT, Harper MJ, Jones IE: **Intensive lifestyle changes are necessary to improve insulin sensitivity: a randomized controlled trial**. *Diabetes Care* 2002, **25**(3):445-452.

31. Lin J: **Robust Modelling and Control of the Glucose-Insulin Regulatory System for Tight Glycemic Control of Critical Care Patients**. Christchurch: University of Canterbury; 2007.
